# Fabrication of Microchannels in a Nodeless Antiresonant Hollow-Core Fiber Using Femtosecond Laser Pulses

**DOI:** 10.3390/s21227591

**Published:** 2021-11-16

**Authors:** Paweł Kozioł, Piotr Jaworski, Karol Krzempek, Viktoria Hoppe, Grzegorz Dudzik, Fei Yu, Dakun Wu, Meisong Liao, Jonathan Knight, Krzysztof Abramski

**Affiliations:** 1 Laser & Fiber Electronics Group, Faculty of Electronics, Photonics and Microsystems, Wroclaw University of Science and Technology, 50-370 Wroclaw, Poland; piotr.jaworski@pwr.edu.pl (P.J.); karol.krzempek@pwr.edu.pl (K.K.); grzegorz.dudzik@pwr.edu.pl (G.D.); krzysztof.abramski@pwr.edu.pl (K.A.); 2 Centre for Advanced Manufacturing Technologies (CAMT), Faculty of Mechanical Engineering, Wroclaw University of Science and Technology, 50-370 Wroclaw, Poland; viktoria.hoppe@pwr.edu.pl; 3 Key Laboratory of Materials for High Power Laser, Shanghai Institute of Optics and Fine Mechanics, Chinese Academy of Sciences, Shanghai 201800, China; yufei@siom.ac.cn (F.Y.); wudakun@ucas.ac.cn (D.W.); liaomeisong@siom.ac.cn (M.L.); 4 Hangzhou Institute for Advanced Study, University of Chinese Academy of Sciences, Hangzhou 310024, China; 5 Centre for Photonics and Photonic Materials, Department of Physics, University of Bath, Claverton Down, Bath BA2 7AY, UK; j.c.knight@bath.ac.uk

**Keywords:** antiresonant hollow core fibers, femtosecond laser micromachining, microchannel fabrication, microstructured fibers

## Abstract

In this work, we present femtosecond laser cutting of microchannels in a nodeless antiresonant hollow-core fiber (ARHCF). Due to its ability to guide light in an air core combined with exceptional light-guiding properties, an ARHCF with a relatively non-complex structure has a high application potential for laser-based gas detection. To improve the gas flow into the fiber core, a series of 250 × 30 µm microchannels were reproducibly fabricated in the outer cladding of the ARHCF directly above the gap between the cladding capillaries using a femtosecond laser. The execution time of a single lateral cut for optimal process parameters was 7 min. It has been experimentally shown that the implementation of 25 microchannels introduces low transmission losses of 0.17 dB (<0.01 dB per single microchannel). The flexibility of the process in terms of the length of the performed microchannel was experimentally demonstrated, which confirms the usefulness of the proposed method. Furthermore, the performed experiments have indicated that the maximum bending radius for the ARHCF, with the processed 100 µm long microchannel that did not introduce its breaking, is 15 cm.

## 1. Introduction

The main goal of laser-based gas spectroscopy research is the development of sensors with high sensitivity and selectivity, preferably maintaining a non-complex design of their setup [1,2,3]. According to the Beer–Lambert law, the detection ability of such sensors depends mainly on the length of the interaction path between the light beam and the measured gas molecules [4]. Hence, long optical paths implemented within the sensors setup are highly desired, especially in the case of low-concentration (typically single parts-per-million (ppm) or below—parts-per-billion (ppb) levels) gas molecules detection. Usually, long optical paths are realized by bulk-optics-based multipass cells (MPCs) that indeed fulfill this crucial requirement, however, at the expense of reduced stability of the sensor due to possible opto-mechanical drifts and optical fringes contributing to the overall noise level. Recent reports demonstrate the use of hollow-core fibers (HCFs), especially the relatively new type of such fibers, ARHCFs, as an alternative to sensors based on MPCs [5,6,7,8]. A gas-filled ARHCF can form a versatile absorption cell characterized by a small volume and can be tailored to a desired length with respect to the specific application. The filling process of the ARHCF core with the target gas mixture can be realized by either gas diffusion through both fiber ends or via specially designed gas cells located at both fiber ends, through which the gas is delivered to the hollow core using overpressure. The gas filling time can vary from several hours per meter for free gas diffusion [9,10] to a few seconds for pressure-supported methods [11,12,13]. A purely diffusion-based gas exchange approach is less complex; however, it significantly increases the response time of an ARHCF-based gas sensor. On the other hand, the use of overpressure filling via additional gas cells increases the sensor complexity and can negatively affect the long-term stability of the sensor. Hence, an alternative solution that will enable noninvasive, simple, and efficient gas filling of the ARHCF core is highly desired.

An alternative method of filling the ARHCF core with the target gas can be realized via side incisions that allow direct access to the fiber air core along the entire length of the fiber. This solution allows non-complex gas exchange without interfering with the optical coupling at the fiber end facets [14]. Due to the fragility of the material, noncontact processing methods are most often used. The most popular method is based on the usage of a femtosecond (fs) laser. For example, a V-shaped hole was made in an HCF [15,16] using the layer-by-layer scanning method. In [17], the researchers presented fabrication of a side hole in an HCF using an fs laser, where the process was carried out in a fluid with a matching refractive index to reduce the debris that is generated during the laser ablation process. The fabrication of microchannels with the use of femtosecond laser pulses introduces certain transmission losses, which mostly result from local damage of the fiber structure or the presence of debris generated during laser ablation.

Another noncontact method used for the production of microchannels is based on the use of a focused ion beam (FIB) as presented in [18,19] for which, due to lack of destructive factors, no losses resulting from the fabrication of the microchannel were recorded. The disadvantage of this method is the time required to process a single hole (~30 min for a single hole with a diameter of 10 µm [19]). Furthermore, because of the limited space in the FIB-based setup, it is impossible to process long (i.e., several meters) fibers in multiple places, which is required for providing optimum gas filling conditions within the long optical path provided by the fiber. For this reason, an attempt was made to find a way to improve the quality of microchannels produced using laser micromachining.

The methods of microchannel realization using an fs laser presented in the literature so far have introduced relatively high losses at the level of 0.45 dB per hole [20], and more. The lowest loss of 0.35 dB per hole was recorded for a single microchannel made in an HCF assisted by liquid [17]. These results are not satisfactory from the potential applications viewpoint since in the case where a higher number of holes is required, the total losses will effectively limit light propagation in such fiber. The most common cause of losses are local modifications of the fiber structure due to laser ablation, leading to an increased attenuation of the propagating light. An additional factor that increases losses is the debris that is formed during the process and is deposited inside the fiber. Both of these factors are the result of the non-optimized laser micromachining process used for modifying the HCF structure.

In this work, we propose a new approach based on the use of the fs laser micromachining process, enabling damage-free and efficient processing of the outer cladding of nodeless ARHCFs. To the best of our knowledge, this is the first attempt to utilize fs laser pulses to access the air core of nodeless ARHCFs via side-drilled microchannels. The developed process does not affect the cladding capillaries that define the low-loss guidance properties of this particular fiber. Such a modification in the outer structure of the ARHFC fiber, as shown in the literature [21,22,23], should not introduce additional transmission losses since the outer cladding has a negligible impact on the light confinement in the air core. With a properly optimized laser-micromachining process, the gap between the capillaries can be used to directly access the hollow region of the microstructured fiber. The key element of the proposed method is the precision of laser processing and proper selection of process parameters in order to significantly reduce the formation of the debris that can accumulate in the air core of the fiber. Minimization of the debris is crucial as it significantly influences the guidance properties of ARHCFs. Here, we will compare two approaches of fabricating lateral channels in ARHCFs in terms of amount of debris, channel surface quality, and processing time. The influence of the microchannel dimensions and bending of the processed fiber will be experimentally verified.

## 2. Materials and Methods

### 2.1. Antiresonant Hollow-Core Fiber

**Figure 1:**
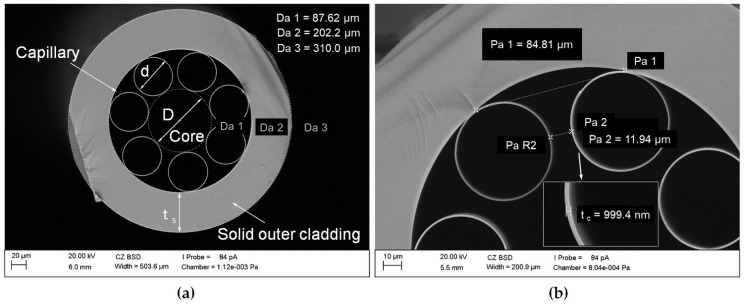
Scanning Electron Micrograph (SEM) cross-sectional image of the ARHCF: (**a**) view of the ARHCF with an air core of 87 µm and (**b**) view of the capillaries forming the fiber cladding.

The ARHCF used for the experiments was fabricated from high purity fused silica glass (Suprasil F300) using the stack and draw technique [24]. The outer diameter of the ARHCF was D_a_ ~310 µm, and the thickness of the solid outer cladding was t_s_ ~54 µm. The hollow core of the ARHCF with a diameter of D ~87.6 µm was defined by the fiber cladding consisting of seven non-touching, circular capillaries with a diameter of d ~54 µm. The distance between the capillaries was between 10 and 12 µm, due to irregularities during the drawing process. The ARHCF was covered with an acrylate polymer layer with a thickness of ~120 µm to minimize mechanical damage factors. Figure 1 shows a cross-section of the fiber used in the experiments (the polymer coating was removed for the fiber cutting process).

According to the ARROW (Antiresonant Reflecting Optical Waveguiding) model [25], the guided wavelength range in an ARHCF is mainly defined by the thickness of the core-wall (capillary). For the fabricated fiber, the thickness of the core-wall was t_c_ ~1 µm, which enabled low-loss transmission of light in both near- (NIR) and mid-infrared (MIR) spectral bands, in the vicinity of 1.5 and 3.34 µm, respectively [12].

### 2.2. Methodology

**Figure 2:**
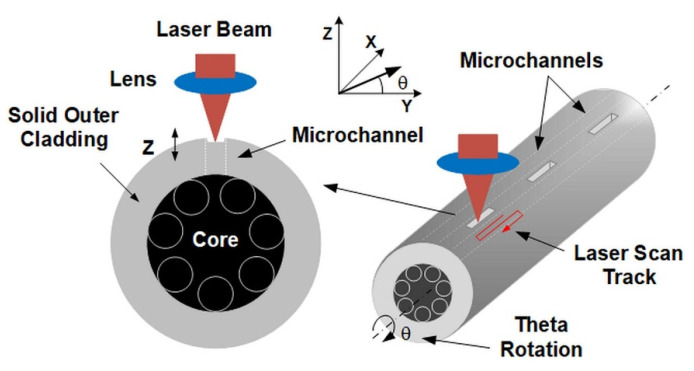
Methods used to process the lateral channels in the outer cladding of the ARHCF.

The method of fabricating the microchannels in the outer cladding of the fiber is shown in Figure 2. The developed laser micromachining process allows for non-contact and damage-free processing of the fragile fiber structure with the required high accuracy and precision. At the point of impact of a high-energy fs laser beam, the material evaporates, leading to the formation of a crater, the depth of which depends on the selected process parameters, i.e., pulse duration, repetition rate, scan speed, pulse energy, and the number of consecutive scans across the processed area. The key element of the method was the precise positioning of the fiber in such a way that the laser beam moved in the area between the inner capillaries, which in our case were 10–12 µm apart. For this reason, it was necessary to control the position of the fiber, which was realized using a CMOS (Complementary Metal-Oxide-Semiconductor) camera and precise motorized XYZ translation stages and theta rotation.

The critical point in the laser processing of such fibers is obtaining a contamination-free microchannel in the outer cladding of the fiber. There is a risk of contamination of the air core by debris generated during the process, which is composed of the glass material that did not evaporate during the process. Any damage to the inner capillaries should also be avoided, as it directly leads to local changes in the resonance properties of this fiber. For this reason, the cutting process should be performed layer by layer so that debris does not penetrate the core and no damage to the capillaries is present.

### 2.3. Experimental Setup and Procedure

**Figure 3:**
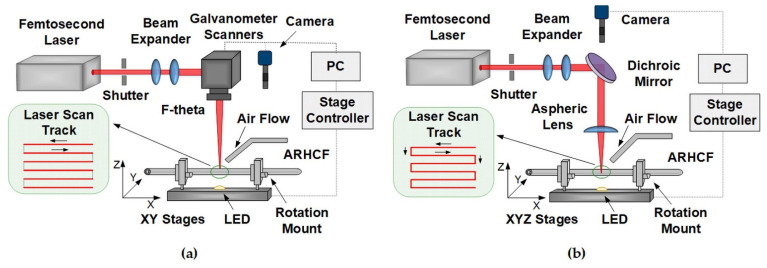
Schematic of the experimental setup for: (**a**) a system with galvanometer scanners and an F-theta lens and (**b**) a moving field and an aspherical lens.

The experiments were carried out using a TruMicro 2030 laser (Trumpf Laser GmbH, Schramberg, Germany), generating fs pulses with adjustable duration from 270 fs to 20 ps, at a wavelength of 1030 nm, an average power of 20 W, and adjustable repetition rate from 200 kHz (100 µJ pulse energy) to 2 MHz (10 µJ pulse energy). Furthermore, the laser has a built-in “pulse picker” that allows operation at selected frequencies (>1 kHz). In order to develop the most effective way of making the lateral cuts in terms of quality and time of implementation, two configurations of delivering the laser beam to the material surface were investigated. In the first approach, shown in Figure 3a, the laser beam, after passing through the beam expander, was directed to the biaxial galvanometer scanners (model IntelliSCAN 14, ScanLab, Munich, Germany) controlled by the TruTops PFO software (Trumpf Laser GmbH, Schramberg, Germany), which was used for fast and precise positioning of the mirrors to deflect the laser beam. The beam (1/e^2^ diameter of 10 mm) leaving the scanner was focused on the sample using an F-theta lens with a focal length of f = 56 mm, producing a focused spot with 1/e^2^ diameter of ~12 µm. Galvanometer scanners in combination with the F-theta lens allowed for rapid scanning of the surface of the processed material (with speeds of m/s or greater), which significantly reduced the time of the process. To ensure precise positioning of the ARHCF with respect to the incident beam, the fiber was placed in a rotating holder (model CRM1, Thorlabs GmbH, Bergkirchen, Germany) mounted on biaxial motorized stages (model 8MTF-102, Standa Inc., Vilnius, Lithuania). Visual control of the process was achieved using a CMOS camera (ALPHA1080 Series, Hangzhou, China).

The second variant of laser micromachining is a system with a moving field of work as depicted in Figure 3b, where the beam, after passing through the beam expander, is focused on the fiber using an aspherical lens (model AL1210-B, Thorlabs GmbH, Bergkirchen, Germany) with a focal length of 10 mm and NA = 0.55, providing a focused spot diameter. ~2 µm (1/e^2^). The fiber placed in a rotating holder (model CRM1, Thorlabs GmbH, Bergkirchen, Germany) embedded in the platform was moved in relation to the incident laser beam using precise motorized XYZ stages at a speed of ~1 mm/s. All three stages were driven by linear motors on the X-Y (model 8MTF-102, Standa Inc., Vilnius, Lithuania), Z axis (model 8MT175-50, Standa Inc., Vilnius, Lithuania), and the positioning resolution for each axis was 0.31 µm. The advantage of this system is the possibility of using the CMOS camera on the same axis as the laser beam, which enables real-time monitoring of the laser ablation processes.

To effectively reduce the occurrence of debris in the laser treated area for both configurations, experiments were conducted in a natural environment without the use of shielding gases except for the transverse airflow used to remove ablation residues.

The research work began with the precise alignment of the fiber in relation to the incident laser beam. For this purpose, a CMOS camera with a source of light transmitted through the fiber, situated perpendicularly to the top layer of the fiber, and a rotating holder were used. By focusing the camera on the surface of the inner capillaries and rotating the fiber with the rotating holder, the tangent points of the inner capillaries to the outer cladding were controlled so that their positions were on the same level. Thus, the space between the capillary pair was centrally located just below the focus area of the laser beam, as shown in Figure 2. In the next step, the location of the focus of the laser beam in relation to the fiber surface was determined. This was achieved by intentionally positioning the fiber far below the focal length of the lens and subsequently moving the fiber mounting platform on the Z axis upward in 2.5 µm steps. During this process a single line scan across the ARHCF was performed at low pulse energy, until the effects of laser ablation were observed (polymer coating was locally mechanically removed using a scalpel before the laser processing). After the location of the focal point was defined with respect to the position of the fiber, subsequent experiments were conducted. The laser cutting is a complex process dependent on several variable parameters related to both the laser source (i.e., pulse energy, repetition rate, average power) and the process itself (e.g., scanning speed, distance between lines—hatching, multiplicity of the process, or a change of the lens focus position in relation to the material). We chose manufactured microchannels with dimensions 250 × 30 µm, which we found to be optimal for the ARHCF used in the experiments (due to the distance between the capillaries of ~12 μm as shown in Figure 1).

In order to define the optimal parameters for the process of cutting a microchannel and the pattern of scanning the surface, a series of systematic experiments were carried out for each setup configuration. A summary of the individual parameters used during the experiments is presented in Table 1. Additionally, we investigated the possibility to remove the protective polymer layer from the fiber with the use of fs laser pulses for both techniques.

After laser treatment, the macroscale analysis of the quality of the microchannels was performed using a digital microscope (model VHX 5000 Keyence, Osaka, Japan) with transmitted light illumination of the sample. Detailed analysis was carried out using the scanning electron microscope (SEM, model EVO MA 25, Zeiss, Oberkochen, Germany). To minimize the charging effect, the fiber was coated with a ~5 nm gold layer using a sputter coater (model Quorum Q150R ES, Lewes, UK). Moreover, the fiber was placed on top of a conductive carbon strip attached to a grounded sample holder.

To investigate the influence of the fabricated microchannels on transmission losses we used a custom-built difference frequency generation-based optical frequency comb source (DFG COMB) operating within the fundamental transmission band of the fiber (3.3–3.4 µm). The broadband comb source was coupled into the ARHCF, and the losses were estimated by measuring the difference in the exiting optical power before and after laser processing of the microchannels. Measurements were taken with the aid of a digital optical power meter (PM100D with high-resolution thermal power sensors S401C, Thorlabs GmbH, Bergkirchen, Germany). This setup was also used to measure the ARHCF bending losses (see Section 3.3 for further details).

## 3. Results

**Figure 4:**
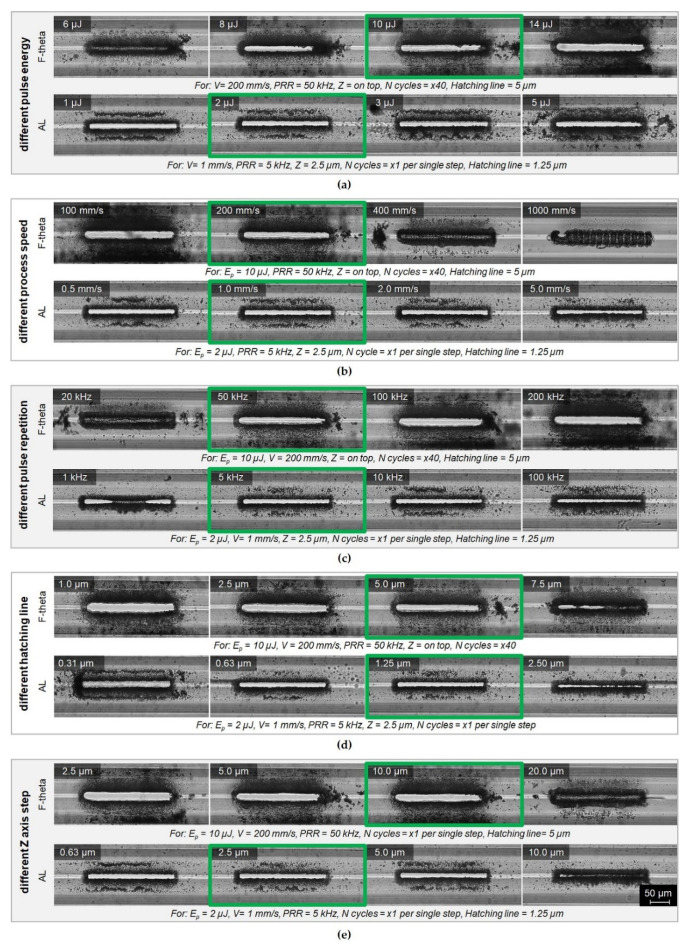
Microscope photographs of microchannels fabricated with the aid of a laser using a galvanometer scanner with an F-theta lens—F-theta and a setup based on a moving field and an aspherical lens, AL. Results are presented for varying parameters: (**a**) pulse energy, (**b**) scanning speed, (**c**) pulse repetition frequency, (**d**) different hatching lines, and (**e**) the Z-axis step of the beam focus position.

Figure 4 presents the selected examples of laser cutting of microchannels in the solid outer cladding of the ARHCF. Photographs of the fabricated microchannels were compared in five sections according to different process parameters used during the laser ablation process. The main criterion for assessing the laser cutting process was the quality of the cut and the efficiency of the process. Qualitative analysis was carried out on the basis of the number and size of microchannels, the occurrence of microcracks in the processing region of the material and the amount of debris generated during the process. Comparison of the quality of the laser-processed microchannels has indicated that the structures made with the use of the F-theta lens are characterized by greater debris in the area of laser treatment, both on the surface and in the air core of the ARHCF. On the other hand, the aspherical lens-aided process introduced significantly less debris and provided a higher quality of the machined features in the fiber structure. In addition, incisions made with the F-theta lens exhibited microcracks that resulted from the generation of high stresses due to the interaction of the high-energy laser beam over a much larger area than in the process where an aspherical lens was used. When analyzing the execution time for the microchannels fabricated by using the F-theta lens, significantly shorter time was achieved (less than one minute) as compared to the process completed using an aspherical lens, where the average time of a laser cutting was in the range between 4 and 15 min per microchannel (depending on the parameters used). Such large differences in the processing time resulted from different diameters of the laser beam and the speed of the processes used.

On the basis of the microscope photographs analysis, the process parameters giving the best results are indicated in green. For the AL-based configuration the optimal parameters were: pulse energy 2 µJ, speed 1 mm/s, repetition frequency 5 kHz, distance between lines 1.25 µm (hatching) and increment along the Z axis with a 2.5 µm step. With these parameters, a single microchannel was fabricated with dimensions equal to 250 × 30 µm in 7 min. For the F-theta configuration, the following parameters were found to be optimal: pulse energy 10 µJ, speed 200 mm/s, repetition ratio 50 kHz, distance between lines 5 µm (hatching) and increment in the Z axis with a step of 10 µm. The optimal parameters result in a total processing time equal to 30 s. After optimization of the processing parameters, both methods of fabricating the microchannels resulted in acceptable quality. The air core of the ARHFC was exposed with the desired microchannel cross-section, no capillaries were damaged, no microcracks in the outer cladding were present, and low accumulation of debris was observed inside the fiber.

**Figure 5:**
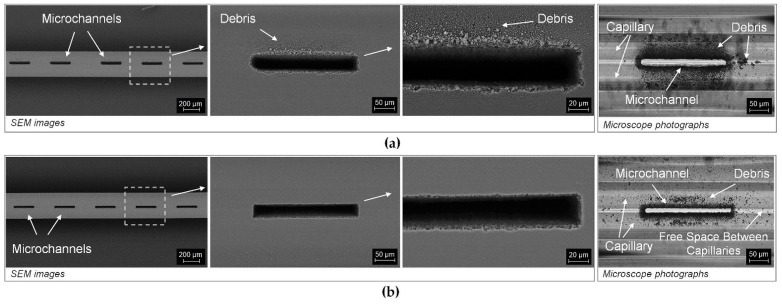
SEM images and microscope photographs of fabricated microchannels using fs laser pulses: (**a**) for the configuration with the F-theta lens and (**b**) for the AL configuration.

Figure 5 shows the results of the fabrication of the microchannels in the ARHCF using both the AL and F-theta setup for the optimized parameters. In both cases, unobstructed microchannels were created along their entire length. The SEM images shown in Figure 5b clearly show that the AL approach delivers a superior overall quality of the fabricated microchannels compared to the F-theta lens-based approach. It can be seen that the amount of debris accumulated in the laser cutting area is negligible, and the edge of each microchannel is very sharp with no visible chipping of the glass material. This is due to the fact that the processing of materials using a small diameter laser beam (~2 μm 1/e^2^) allows the material to be removed in a more controlled manner—layer by layer with a small increment in the Z axis, however, at a cost of increased process duration. A microscope photography of the fabricated microchannel taken with visible light illumination clearly shows the differences in the quality of the fabricated features.

**Figure 6:**
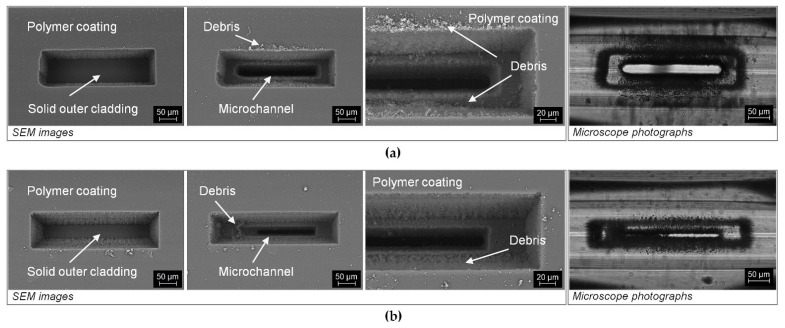
SEM images and microscope photographs of the polymer coating removal process and the laser microcutting of the microchannel realized in one process: (**a**) with the F-theta configuration and (**b**) with the AL configuration.

Figure 6 shows the results of the experiments during which the microchannels were fabricated without prior removal of the outer polymer layer. This approach eliminates the requirement of stripping the polymer coating of the fiber using, e.g., a scalpel blade, which is not straightforward and can easily damage the fiber, especially in the case when several tens of meters long fiber need to be laser-processed in the middle of its length. For both processing setups, the polymer layer was successfully laser-removed during the process before ablating the glass-based outer clad of the fiber. This method of selective laser removal of the polymer layer is superior when compared to preprocessing mechanical removal from a larger, neighboring area. The laser-based method is ablating the polymer directly above the microchannel area, which does not weaken the overall structure of the fiber as much as pre-processing removal of the fiber. The parameters used for polymer removal with the fs laser were identical as those for the microchannel cutting, except that for the AL method the number of steps on the Z-axis was increased to fully remove the polymer layer. Analyzing the results obtained for the F-theta lens, a much more efficient removal of the polymer was observed (Figure 6a) than for the AL system. This is a direct result of using a larger beam, which resulted in evaporation of the material from a larger surface for a single laser pulse. Moreover, it was observed that the exposure of the outer optical cladding in a small area causes disturbance of the air flow used to remove the free fractions of the debris from the site of interaction of the laser beam with the fiber (polymer layer thickness ~120 μm), which is visible in the case of the AL technique (Figure 6b). As a result, the debris that arose during the polymer coating removal was deposited in the crater formed during laser cutting, effectively blocking the laser ablation process and directly leading to the partial free-flow fabricated microchannel. The use of vertical air flow in this case would mitigate the problem, but there is a risk of blowing the generated debris into the ARHCF core, which would have a negative impact on the transmission properties of the fiber, in the worst case causing severe losses. Therefore, the proposed solution to this problem is based on material removal from a larger area to ensure improved external air flow at the bottom of the microchannel being processed.

### 3.1. Influence of Laser Microchannel Processing on the ARHCF Transmission Characteristic

**Figure 7:**
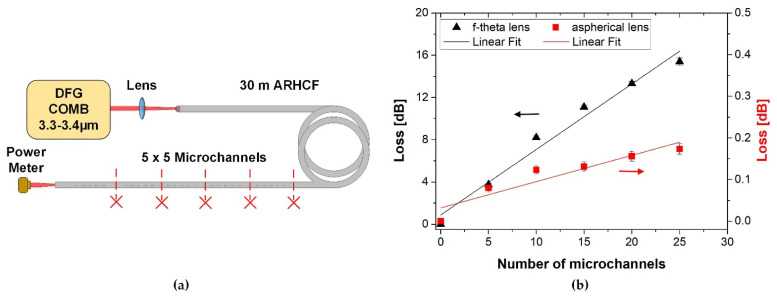
Influence of microchannel fabrication on transmission loss in the ARHCF for wavelengths 3.3–3.4 µm: (**a**) experimental setup and (**b**) loss measurement calculated as a function of the number of microchannels.

To verify to what extent the fabricated microchannels affect the transmission properties of the ARHCF, an experiment was performed in which a set of microchannels were manufactured with selected processing parameters. In this stage, a 30 m long ARHCF was used and 5 sections with 5 microchannels each (250 × 30 µm) were manufactured. The distance between the microchannels was 1 mm and the separation between the sections was equal to 10 cm. A diagram of the experimental setup is presented in Figure 7a. A custom-built DFG COMB was used as a broadband light source operating in the 3.3–3.4 µm wavelength region, matching a part of the low-loss transmission band of the ARHCF. The loss introduced by the fiber modification was calculated by comparing the optical power delivered by the unprocessed fiber and the fiber with laser-machined microchannels. First, the fiber-delivered power was measured for the ARHCF with 5 sections of microchannels processed at its end. Subsequently, one section of the microchannels was cut off from the fiber using a ceramic blade, and the measurement of the delivered optical power was performed again. This procedure was repeated for each section until the entire processed part of the fiber was removed. Note that during the measurements the input light coupling conditions were maintained the same for each step, so the average optical power of the DFG COMB radiation coupled into the ARHCF core was constant. The results obtained are presented in Figure 7b.

The microchannels produced with the F-theta lens introduced significantly greater transmission losses (black triangle) compared to the lateral cutting made with the aspherical lens (red square). For example, for the 25 microchannels made with the F-theta lens, the loss was 15.49 dB, while for the aspherical lens it was only 0.17 dB. Based on theoretical calculations presented in [21,22,23], the introduction of lateral cuts in the outer cladding of the nodeless ARHCF should not lead to significant transmission deterioration since the outer cladding does not participate directly in guiding the light inside the core. To investigate the cause of the increase in loss, a detailed analysis of the fabricated microchannels was carried out.

**Figure 8:**
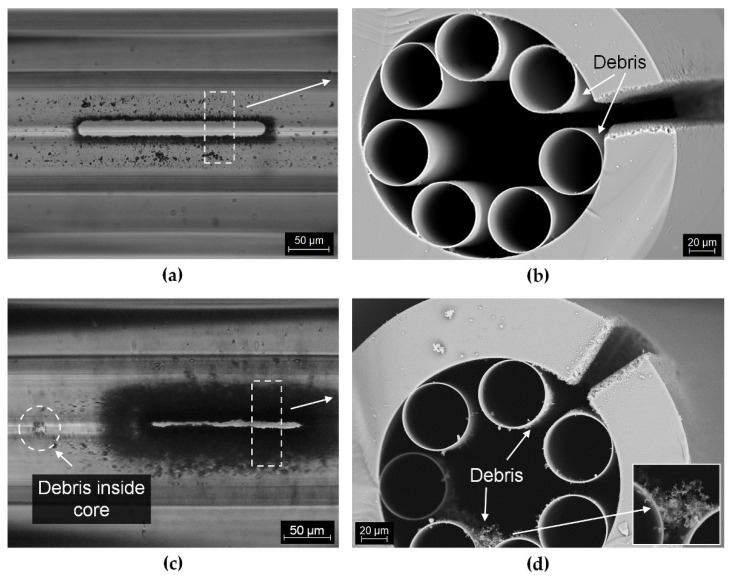
Images of microchannels processed in the ARHCF: (**a**) using an aspherical lens, (**b**) SEM cross-sectional image with debris in the vicinity of the microchannel made using an aspherical lens, (**c**) using the F-theta lens, and (**d**) SEM cross-sectional images with debris in the vicinity of the microchannel made with the F-theta lens.

Figure 8 shows photographs of randomly selected microchannels made with the aid of the aspherical lens (Figure 8a,b) and the F-theta lens (Figure 8c,d). In both cases, the microchannels were made in a region exactly above the free space between the inner capillaries, without damaging their structure. The photographs taken with visible light illumination show the presence of impurities inside the fiber within the manufactured microchannels only in the case of using the F-theta lens approach. By analyzing the SEM images of the fiber cross-sections (Figure 8b,d), we can conclude that for the microchannels made with the use of an aspherical lens, only a low degree of impurities occurs in the space between the outer cladding and the capillaries. For microchannels made with the F-theta lens, the amount of impurities inside the fiber is much greater (Figure 8c,d). The formation of large particles, which settle on the walls of the capillaries forming the core, is visible not only in close proximity to the lateral cut, but they also displace into the inner section of the air core, for example due to the pressure difference, which is shown in Figure 8c. The presence of such large fractions leads to deterioration of transmission in the fiber, due to scattering effects, attenuation of the glass particles at wavelengths longer than 2.5 µm and as a result of influencing the ARHCF guiding mechanism, which is directly dependent on the thickness of the capillaries. The formation of debris is directly related to the laser ablation process, during which the material is removed by evaporation. The condensation of vapors induced by the collision with cooler gas molecules from the environment leads to the deposition of the debris in the vicinity of the laser beam or on the walls of the formed crater. This leads to a deterioration in the effectiveness of laser ablation in the next cycle of the process [26,27,28]. Therefore, the increased amount of debris in the case of laser processing with the F-theta lens results mainly from the processed surface area (due to the relatively large focused spot size) from which the material is evaporated in a short time. With this in mind, further experiments were performed using an aspherical lens for which; due to almost an order of magnitude smaller focused beam diameter on the sample and the speed of the process, the amount of debris was significantly reduced.

### 3.2. Influence of Microchannel Length on the ARHCF Transmission

**Figure 9:**
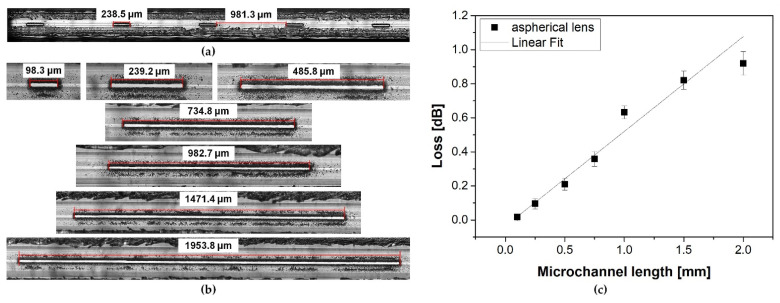
Additional loss of the ARHFC plotted as a function of the length of the microchannel: (**a**) view of a single section containing 5 microchannels with a length of 0.25 mm, (**b**) microchannels with lengths equal to 0.1, 0.25, 0.5, 0.75, 1, 1.5, 2 mm, respectively, and (**c**) loss vs. microchannel length.

To determine the usefulness of the proposed method for modifying the ARHCF structure with the aid of the ultrafast laser micromachining process, we have investigated the possibility of cutting long microchannels with a length of up to ~2 mm. Here, 35 microchannels were made at the output end of a 15 m-long ARHCF (the same fiber as in the previous experiment) in 7 sections, each containing 5 microchannels with different lengths and fixed widths equal to 30 µm. The distance between the sections was 10 cm and the microchannels were separated by 1 mm. The polymer coating was removed locally, mechanically using a scalpel prior to laser processing. All microchannels were made using an aspherical lens for optimal process parameters, as defined at the beginning of this section. Figure 9a,b shows fabricated microchannels with lengths of 0.1, 0.25, 0.5, 0.75, 1, 1.5 and 2 mm. The quality of the fabricated microchannels was at the same level regardless of their length. In-depth analysis of the fabricated structures has shown that no damage occurred to the internal capillaries that form the actual fiber cladding. The impact of a microchannel length on the transmission properties of the ARHCF was determined using a similar approach to that previously described in Section 3.1. The optical power at the fiber output was measured after individual sections of the microchannels were cut off. The results are plotted in Figure 9c. It can be seen that microchannels with a length below 0.5 mm introduce very low transmission loss, not exceeding 0.1 dB. For microchannels with lengths greater than 0.5 mm, an increase in light attenuation was observed. The higher loss level observed in the case of longer microchannels was caused by the accumulation of debris in the vicinity of the processing area, which was pushed into the core by the air flow used to clean the processed region.

### 3.3. Bending Performance of the ARHCF with Manufactured Microchannels

**Figure 10:**
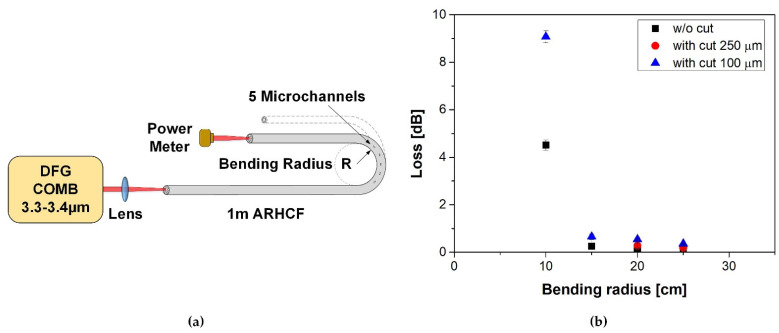
Bending loss of the ARHCF with microchannels: (**a**) schematic of the measurement setup and (**b**) results of measurements plotted as a function of the bending radius.

From an application point of view, an interesting aspect is determining to what extent the fabrication of lateral microchannels will affect the strength of the ARHCF and its transmission. For this purpose, an ARHCF with a length of 1 m was laser processed in its midsection to fabricate 5 microchannels with two different lengths of 100 and 250 µm and a width of 30 µm. Microchannels were produced with the use of an AL configuration and using the parameters defined at the beginning of Section 3. Figure 10a shows a schematic of the measurement system, where the fiber was bent at radii equal to 10, 15, 20 and 25 cm. Microchannels did not change their location relative to the plane during the shift of bending radius. The additional loss introduced by the modification of the fiber structure was defined by comparing the optical power transmitted through an unprocessed fiber and a fiber with the fabricated microchannels with different bending radiuses. The results are shown in Figure 10b.

The performed experiments indicated that for a 100 µm long microchannel (blue triangles), the bending-related loss is comparable to an unprocessed fiber for bending radius in the range between 15 and 25 cm. When the bending radius was reduced to 10 cm, a significant increase in loss (~9 dB) was observed, which was caused by a crack in the outer optical cladding within one of the microchannels. For 250 µm long microchannels (red dots), low-loss transmission was observed for a bending radius in the range of 20 to 25 cm. The lower bending radius resulted in permanent damage (fracture) to the fiber directly in one of the fabricated microchannels. This can be attributed to a significant weakening of the ARHCF structure in the processing region.

## 4. Discussion

In this work, we have demonstrated the method of fabricating microchannels in an ARHCF based on the laser ablation process. Conducting detailed research on the strategy and process parameters allowed for reproducible fabrication of microchannels in the ARHCF, which introduced relatively low losses (<0.01 dB for a single microchannel). So far, in the literature, the lowest loss for a single microchannel made with an fs laser on an HCF fiber was 0.35 dB [17]. In comparison, this result is over 35 times worse than the result obtained using the method developed within our research. In another article, a 20 µm microchannel made by laser ablation introduced 1 dB loss [29]. In both cited cases, the holes were made in the hollow-core photonic bandgap fiber (HC-PBG). Due to the complex structure and the light-guiding mechanism in HC-PBG, during the fabrication of microchannels interference in the structure of the fiber is inevitable through microdefects that locally disturb the periodicity and symmetry of the fiber, leading to a deformation of the PBG structure, and hence increase in the transmission loss of the fiber. Recently, there was a report about the creation of a 150 µm long channel using an fs laser in a hollow core negative curvature fiber (HC-NCF), which in its structure is similar to the fiber used by us, however, its cladding structure is formed by a set of non-gapless capillaries. The channel fabricated in the HC-NCF introduced a 0.45 dB loss, which mainly resulted from the microdestruction of the cladding capillaries, which are responsible for confining the light into the air core of the fiber. Table 2 summarizes the best results obtained for single microchannels made in the laser ablation process in HCFs.

The fabrication of a microchannel that showed a low loss (<0.01 dB) partly results from the relatively non-complex structure of the fiber used by us. The mechanism of light guidance in ARHCF takes place within the air core limited by internal capillaries, and interference with the outer cladding by the fabrication of microchannels of different lengths does not have a significant impact on the optical performance of the fiber, which has already been described in detail in [30]. In our case, the gap between the capillaries constituted a natural part of the channel, for which it was not necessary to introduce changes to the fiber structure. Therefore, the key element of the process was the precision of guiding the laser beam so that, in the final stage of the implementation of the microchannel in the outer cladding, the internal capillaries would not be accidentally damaged. The second important factor was the amount of debris formed during the laser ablation process, which, after the process was conducted, was found both outside and inside the ARHCF. It was possible to reduce the problem related to formed debris to some extent by means of an appropriate correlation of the applied process parameters: scanning speed, radiation power and pulse repetition rate, or spot size of the laser beam. An additional advantageous element was the use of air blown parallel to the fiber plane during the process. In this way, the debris generated during laser ablation was to some extent blown off the surface of the treated fiber, without causing excessive accumulation and priming of the laser beam. Obtaining low losses for single microchannels fabricated in the ARHCF allows us in future research to use them in a greater number to significantly accelerate the diffusion of gas into its interior.

In most of the works, the fabricated microchannels were realized for relatively short sections of the fiber (up to several centimeters) and their analysis was limited only to the losses they introduced without addressing the issue of weakening the fiber structure and lowering mechanical resistance. In this work, it was shown that while maintaining precision and limiting the amount of generated debris, it is possible to fabricate microchannels from single µm to several mm. The height of the microchannels in this case was limited to the free space between the capillaries, which was 10–12 µm at the narrowest point. In addition, studies were carried out to determine to what extent the fabrication of microchannels affects the strength of the fiber and bending resistance, which for potential applications in the construction of long absorption cells or sensors is important in their miniaturization. It has been proven that when bending is required, it will be more advantageous to fabricate microchannels having a relatively short length, since they exhibit greater bending resistance due to the shorter length of the fiber subjected to modification.

## 5. Conclusions

We have demonstrated an efficient method of producing microchannels in a nodeless ARHCF using an fs laser. The development of optimal process parameters and a laser cutting strategy allowed the fabrication of microchannels in the outer cladding of the ARHCF without damaging the internal capillaries forming the antiresonant structure. The key element of the proposed technology was to carry out the process in a way that allowed us to minimize the formation of the debris that occurred during laser ablation. For a single microchannel, there was a slight increase in transmission losses (<0.01 dB), which is more than 35 times lower than reported so far [13]. Moreover, the implementation of microchannels of different lengths did not cause a significant increase in losses, which proves that in the case of ARHCF it is possible to make microchannels of any length, provided that the fiber is precisely positioned in relation to the laser beam. Additionally, it has been shown that making lateral incisions in the ARHCF weakens its mechanical resistance to bending. Coiling of the fiber in a small diameter is possible only in the case of short microchannels <0.1 mm, since for longer microchannels the bending forces can fracture the ARHCF in the area subjected to laser modification. The presented work shows that the proposed micromachining process can be used to manufacture microchannels in ARHCFs, thus allowing the construction of low-loss fiber-based absorption gas cells for applications in laser-based gas sensing.

## Figures and Tables

**Table 1:** Laser and process parameters used for optimization of laser cutting of the microchannel.

Parameter	F-theta Lens	Aspheric Lens
Wavelength (nm)	1030	
Pulse duration (fs)	270	
Spot size (1/e^2^) (µm)	~12	~2
Pulse Energy (µJ)	6–14	1–6
Repetition ratio (kHz)	20–1000	1–200
Speed (mm/s)	50–1000	0.5–5
Z move step (µm)	2.5–20	0.5–10
Number of cycles N	1 per step × 10–× 50	1 per step
		-
Hatching line (µm)	1–10	0.5–2.5

**Table 2:** Comparison of the losses induced by microchannels in HCFs.

Type of Fiber	Microchannel Type	Loss (Per Single Channel)	Ref.
HC-PBGF	V-shaped microchannel 20 µm diameter (on top cladding)	1 dB	[29]
SC-HF	V-shaped microchannel 20 µm diameter (on top cladding)	0.5 dB	[16]
HC-PBGF	Hole diameter 1.5 µm (on top cladding)	0.35 dB	[17]
HC-NCF	Slot x-axis—150 µm, y-axis across the fiber	0.45 dB	[20]
ARHCF	Microchannel size x-axis—250 µm, y-axis—30 µm	0.007 dB	[this work]

## Data Availability

Not applicable.
